# Contribution of the latent transforming growth factor-beta binding protein 2 gene to etiology of primary open angle glaucoma and pseudoexfoliation syndrome

**Published:** 2013-02-07

**Authors:** Sahar Jelodari-Mamaghani, Ramona Haji-Seyed-Javadi, Fatemeh Suri, Naveed Nilforushan, Shahin Yazdani, Kambiz Kamyab, Elahe Elahi

**Affiliations:** 1 School of Biology, University College of Science, University of Tehran, Tehran, Iran; 2 Eye Research Center, Rassoul Akram Hospital, Tehran University of Medical Sciences, Tehran, Iran; 3 Ophthalmic Research Center, Labbafinejad Medical Center, Shahid Beheshti University of Medical Sciences, Tehran, Iran; 4 Department of Pathology, Razi Hospital, Tehran University of Medical Sciences, Tehran, Iran; 5 Department of Biotechnology, College of Science, University of Tehran, Tehran, Iran

## Abstract

**Purpose:**

To assess for the first time the possible contribution of latent transforming growth factor (TGF)-beta binding protein 2 (LTBP2), an extracellular matrix (ECM) protein that associates with fibrillin-1-containing microfibrils, to the etiology of primary open angle glaucoma (POAG) and pseudoexfoliation (PEX) syndrome. Mutations in *LTBP2* have previously been shown to be the cause of primary congenital glaucoma (PCG) and other disorders that often manifest as secondary glaucoma.

**Methods:**

All exons of *LTBP2* were sequenced in the DNA of 42 unrelated patients with POAG and 48 unrelated patients with PEX syndrome. Contribution of candidate variations to disease was assessed by screening in control individuals and use of biochemical, bioinformatics, and evolutionary criteria, and in one case by segregation analysis within the family of a proband with POAG. Microscopy was performed on the skin of a patient with PEX syndrome whose condition developed into PEX glaucoma during the course of the study and on the skin of her son previously identified with PCG who harbored the same *LTBP2* mutation.

**Results:**

Among the 30 sequence variations observed in *LTBP2*, five found in five patients with POAG and two found in two patients with PEX glaucoma syndrome may contribute to their diseases. One of the mutations was observed in a patient with POAG and in a patient with PEX glaucoma syndrome. Light, fluorescent, and electron microscopy showed that a mutation present in one of the individuals affected with PEX glaucoma syndrome and in her son affected with PCG causes disruptions in the ECM.

**Conclusions:**

Some *LTBP2* sequence variations can contribute to the etiology of POAG and PEX glaucoma syndrome. It is not expected that in these diseases *LTBP2* mutations behave in a strictly Mendelian fashion with complete penetrance. In conjunction with recent findings, the results suggest that anomalies in the ECM are among the factors that can contribute to POAG and PEX glaucoma syndrome. *LTBP2* and other related ECM protein coding genes should be screened in larger cohorts with these diseases, which are common disorders and important to the public health.

## Introduction

*LTBP2* (OMIM 602091), the gene encoding latent transforming growth factor (TGF)-beta binding protein 2, was identified in 2009 as a gene causing primary congenital glaucoma (PCG; OMIM 231300) [1,2]. More recently, it was shown that *LTBP2* mutations can also cause megalocornea [3,4], microspherophakia [5], and Weill-Marchesani syndrome (WMS; OMIM 277600) and promote Marfan syndrome (MFS; OMIM 154700) features [6]. The possible contribution of *LTBP2* to primary open angle glaucoma (POAG; OMIM 137760) and pseudoexfoliation syndrome (PEX; OMIM 177650) is addressed in this study. *LTBP2* on chromosome 14 encodes an extracellular matrix (ECM) protein that is a member of a superfamily composed of multiple fibrillin and LTBP proteins [7,8]. LTBP2 is expressed in elastic tissues and associates with fibrillin-1-containing microfibrils [9]. It is believed that LTBP2 has functions related to those of microfibrils and elastic fibers. In addition to structural roles, it may affect TGF-β activities.

Glaucoma, the second leading cause of blindness, is a heterogeneous group of optic neuropathies that manifest by optic nerve head cupping or degeneration of the optic nerve, resulting in a specific pattern of visual field loss [10]. Increased intraocular pressure (IOP) is often associated with the condition. The disease is sub-grouped based on etiology, anatomy of the anterior chamber, and age at onset [10]. POAG accounts for 70% of glaucoma cases in Caucasian populations and usually affects individuals past the age of 40 [11]. In this form of glaucoma, the anterior chamber angle appears normal; this form is associated with variable severity and phenotypic expressivity [12,13]. Although POAG in some families demonstrates Mendelian inheritance, the disease usually presents as a complex disease. After identifying *LTBP2* as a PCG causative gene, the authors considered the gene might also cause POAG [1,2]. A POAG locus (LOD scores of 3.99) on chromosome 14q has been reported in a linkage study [14]. Cytogenetic studies have also suggested a tentative link between 14q and glaucoma [15,16]. Furthermore, a quantitative trait locus for high IOP, a prominent feature of glaucoma, has been reported on 14q [17]. In addition, mutations in the PCG causative gene *CYP1B1* have been observed in patients with POAG, and it was thought that other genes including *LTBP2* may also have roles in the etiology of both diseases [18-20]. Finally, all the disorders so far identified that can be caused by mutations in *LTBP2* often manifest secondary glaucoma.

Pseudoexfoliation (PEX) syndrome is a common disorder that usually affects elderly individuals [21]. It is often accompanied by glaucoma and is in fact one of the most common causes of glaucoma. PEX syndrome is considered a fibrotic matrix disorder, characterized by stress-induced elastosis and excessive production and abnormal cross-linking of elastic microfibrils [22]. Aggregates in the form of what is known as PEX material deposit mainly in the anterior segment of the eye. PEX material consists of various elastic and microfibril-related proteins, including fibrillin-1 and LTBP2. Given that PEX is a fibrotic disorder often associated with glaucoma (PEXG) and that LTBP2 is a component of PEX material, the possibility that *LTBP2* mutations may be associated with this disease was also considered.

We performed mutation screening of *LTBP2* in a cohort of 90 patients with POAG or PEX syndrome. To the best of our knowledge, this is the first time *LTBP2* has been screened in patients with these disorders. Sequence variations were found in both types of patients, and the properties of the variations support the proposition that *LTBP2* may have a role in the etiology of these diseases. Microscopic examination of skin fibroblasts of two patients with *LTBP2* mutations showed notable disruption of the ECM. Our results emphasize that the ocular anomalies in some patients with POAG and PEX can be due to disruptions in the ECM.

## Methods

This study was performed in accordance with the Declaration of Helsinki and with approval of the ethics board of the University of Tehran. All participants or their responsible guardians consented to participate after being informed of the nature of the research.

### Subjects

Forty-two and 48 Iranian individuals with POAG and PEX syndrome, respectively, were recruited from the Ophthalmology Department of Labbafinejad Medical Center (associated with Shahid Beheshti University of Medical Sciences) and Hazrat Rassoul Hospital (associated withTehran University of Medical Sciences). The patients did not report symptoms of other disorders at the time of recruitment, except one patient (211L) who reported a cardiac anomaly as described below. None of the 90 patients were related. As will be described, one of the patients with PEX syndrome was the mother of a patient with PCG examined in a previous study [2]. The patients were recruited consecutively, without regard to disease presentation being apparently sporadic or familial and without regard to age at onset. Sex was distributed almost equally in each group of patients. All patients with POAG (range 19-76 year; average: 54) and PEX syndrome (range 62-83 year; average: 76) syndrome were diagnosed by one or two glaucoma specialists (N.N. and S.Y.). Slit-lamp biomicroscopy, IOP measurement, gonioscopy, fundus examination, and perimetry were performed. Perimetry was performed at least two times in all patients with POAG and PEX syndrome harboring putative disease contributing variations. IOP measurements were obtained using Goldmann applanation tonometry. Criteria for diagnosis of POAG were the presence of at least two of the following criteria: an IOP greater than 21 mmHg in at least one eye or inter-eye IOP asymmetry exceeding 8 mmHg; characteristic glaucomatous optic nerve head or retinal nerve fiber layer (RNFL) changes (e.g., vertical cupping, neural rim thinning or loss, RNFL dropout); and visual field defects not attributable to other causes. None of the patients with diagnosed POAG had other ocular anomalies, and all presented with an open anterior chamber angle in the affected eyes. Furthermore, all patients with POAG were screened for mutations in *MYOC* and *CYP1B1* as previously described, and only those without disease-associated mutations in these genes were screened for variations in *LTBP2* [18]. After genetic screening, attempts were made to obtain familial data from the patients with POAG in whom candidate disease-contributing sequence variations were observed.

PEX syndrome diagnosis was based on observation of PEX material on the anterior lens capsule and/or pupillary margin after mydriasis by slit lamp biomicroscopy [23]. Secondary open angle glaucoma in patients with PEX syndrome was diagnosed as described for POAG. Thirty-three (70%) of the patients with PEX presented with secondary glaucoma (patients with PEXG). Four hundred Iranian unrelated control individuals older than 60 years of age without self-reported family history of ocular diseases were also recruited.

### Latent transforming growth factor-beta binding protein 2 gene screening

*LTBP2* exons and flanking intronic sequences were amplified with PCR, and subsequently sequenced with the Sanger protocol [2]. The *LTBP2* reference sequences used were NT_026437.12, NM_000428.2, and NP_000419.1. The effects of the variant sequences on splicing were predicted by using NNSPLICE 0.9, Human Splicing Finder V 2.4.1 and GENSCAN. Potential effects of all variations on splicing, including those that affected amino acid changes, were checked. To determine the extent of the conservation of amino acids altered due to nucleotide variations, the amino acid sequences of homologous proteins from other species were aligned using the ClustalW2 software. Variations deemed to contribute to disease status were assessed in control individuals with allele-specific PCR protocols or restriction fragment length polymorphism analysis. The number of control individuals screened differed for the different variations and ranged from 200 to 400 (Table 1). The structural consequences of these variations were predicted using the PolyPhen-2 in silico tool [24]. The sequences of all primers used to amplify the *LTBP2* exons are available in supplementary Appendix 1.

**Table 1:** Novel sequence variations in LTBP2.

**Mutation**	**Het/** **Homo**		**Protein** **location**		**Change** **in charge** **of aa**		**Change in** **size of R** **group of aa**	**PolyPhen** **prediction/** **HumDiv score**	**Conservation**	**No. controls** **checked/** **presence**	**Comments**	
**POAG**												
p.Pro432Leu	Het		Close to right border (p.428)		^−^		Small>large	Probably	^+ + + +^	400/-	Also observed	
			of 2nd EFG-like motif				(Ring>linear)	damaging/1			in PEXG patient	
p.Arg495Gln	Het		Not within known motif		^+^		Large>medium	Probably	^+ + +^	400/-	Familial data supports	
								damaging/0.999			role in disease	
p.Glu1191Lys	Het		Adjacent to p.Cys1192		^+^		Medium>large	Probably	^+ + +^	400/-		
			of 12th EFG-like motif					damaging/0.987				
p.Gln1417Arg	Homo		Within TB2 motif		^+^		Medium>large	Benign/0.249^§^	^+ +^	400/-		
								
p.Val1638Met	Het		Very close to right border		^−^		Medium>large	Probably	^+ +^	400/-		
			(p.1636) of TB3 motif					damaging/0.998				
**PEX syndrome**												
p.Pro432Leu		Het	Close to right border (p.428)		^−^		Small>large	Probably	^+ + + +^	400/-	Also observed	
			of 2nd EFG-like motif				(Ring>linear)	damaging/1			in POAG patient	
p.Tyr1792fsX55*		Het	Stop codon within							400/-	Causative of PCG	
			cbEFG-like 20 motif								in Hom state	
**Other novel variations probably not disease associated**												**Reason assessed**
												**not disease associated¤**
p.Ser518Ile		Het		Not within known motif		^−^	Very small>large	Possibly	^+^	100/2	Observed in POAG	Also observed
								damaging/0.837			(2X),PEX,	in controls
											simple EL patients	
p.Pro1452Pro		Het		Within TB3 motif		^−^	^−^			ND	Observed	Creates synonymous
											in POAG patient	codon
p.Pro1556Pro		Het		Within cbEFG-like 18 motif		^−^	^−^			ND	Observed	Creates synonymous
											in POAG patient	codon
p.Ile667Leu		Het		Not within known motif		^−^	^−^	Benign/0.028	^+^	200/-	Observed in	Similar amino acid
											PEX patient	in other species
Intronic		Het								ND	Observed in	No effect on splicing
											PEX patient	
p.Met1567Val		Het		Within cbEFG-like motif 18		^−^	Large>medium	Benign/0.000	^+^	200/-	Observed in	Val observed in
											PEXG patient	non-primate species

* Not novel, previously observed in PCG affected sons; § Considered potential risk factor despite HumDiv score because of other features shown in table; ¤ As stated in text, we opted for stringent criteria in predicting possible association with disease status; ^+^: Conserved in primates; ^+^^+^: Conserved in mammals; ^+^^+^^+^: Conserved in mammals & birds; ^+^^+^^+^^+^: Conserved in mammals, birds & fish; ND: not screened in controls; Het: heterozygous,Homo: homozygous.

### Histology

To assess the effect of an *LTBP2* mutation observed in a patient with PEX syndrome and previously observed in related patients with PCG, the ECM of skin fibroblasts from patients harboring the mutation was examined using light, immunofluorescent, and electron microscopy. A skin tissue specimen with a depth of approximately 6 mm was obtained from behind the ears of these patients and age- and sex-matched control individuals. The specimens were cut into portions and placed in glutaraldehyde or tissue freezing medium. One glutaraldehyde-fixed sample from each individual was processed for staining with Orcein Giemsa to stain elastin and with Trichrome to stain collagen. For immunofluorescent staining, cryosections were incubated with goat polyclonal antibody against human LTBP2 (LTBP2 (N20), Santa Cruz Biotechnology, Inc., Santa Cruz, CA) or rabbit polyclonal antibody against human fibrillin-1 (ab53076, Abcam, Cambridge, England). Negative controls were not incubated with primary antibody. The sections were subsequently incubated with fluorescent conjugated secondary antibodies (Santa Cruz Biotechnology). Nuclei in the same sections were counterstained with 4’, 6-diamidino-2-phenylindole (Invitrogen, Carlsbad, CA). Finally, samples were processed for electron microscopy, and 60 nm sections were visualized with a Zeiss EM900 transmission electron microscope (Carl Zeiss, Jena, Germany).

## Results

**Figure 1:**
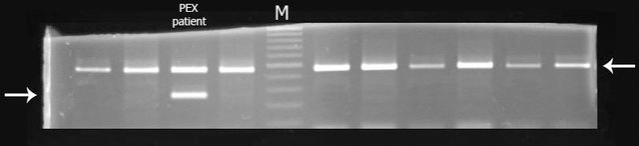
Screening of c.1999A>C mutation by an allele-specific polymerase chain reaction. Polymerase chain reaction (PCR) was performed in presence of two pairs of primers, one for amplification of a 456 bp fragment in *FBN1* and the other for amplification of a 259 bp fragment in *LTBP2*. The former served as control for efficacy of the PCR reaction. The 3′ terminus of the forward primer for the *LTBP2* fragment was designed to amplify only the mutated allele (c.1999C) and not the wild-type allele. M, size markers; patient with PEX, template was from patient with PEX carrying a mutation; template in all other lanes is from control individuals. The arrow on the left shows the migration position of the *LTBP2* product, and the arrow on the right shows migration position of *FBN1* product. Comparable results were obtained in all 100 controls screened.

**Figure 2:**
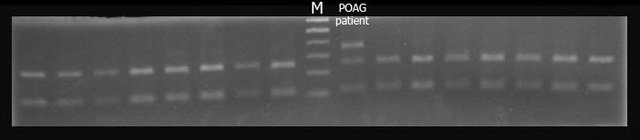
Screening of c.4912G>A mutation by restriction fragment length polymorphism. Polymerase chain reaction products of exon 34 were digested with HpyCH4IV. Three bands are evident in the electrophoretic pattern of mutation carrier (POAG patient), and only two bands in the electrophoretic pattern of the control individual. M, size markers.

**Figure 3:**
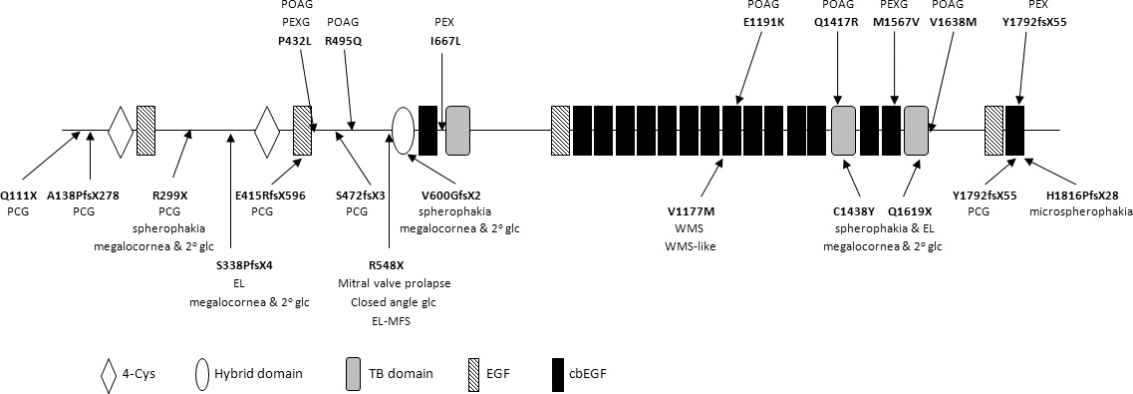
Positions of putative disease-associated mutations in latent transforming growth factor-beta binding protein 2. Positions of disease-associated mutations observed here are shown above the schematic representation of the latent transforming growth factor-beta binding protein 2 (LTBP2) structure, and the positions of the mutations previously reported are presented below the diagram. Diseases associated with the mutations are also given. Symbols used for the various LTBP2 domains are indicated; horizontal lines represent protein regions not known to be specific domains.

Thirty *LTBP2* sequence variations were observed in the 90 individuals screened (Appendix 2). Nineteen are previously reported variations, and 11 are novel variations. The allele frequency of 18 of the non-novel variations as reported in the Single Nucleotide Polymorphism Database (dbSNP) ranged from 0.012 to 0.405. The dbSNP data incorporates data derived from the 1000 Genomes Project. Six of the novel variations were observed in controls, created synonymous codons, were not highly conserved during evolution, and/or were intronic variations predicted not to affect splicing. We opted for a conservative approach and considered that these 24 variations did not contribute to disease status; clearly, variations reported in databases and variations observed in controls may predispose individuals to disease, particularly common and late onset diseases such as POAG and PEX syndrome. A previously identified PCG causative mutation and five novel variations were considered reasonable candidates for being variations or risk factors contributing to POAG and/or PEX. All but one were observed in the heterozygous state. Considerations including position of the affected amino acid within the protein, the biochemical nature of the amino acid change, the bioinformatics prediction of the effect of the change, the extent of the evolutionary conservation, and absence in controls were taken into account in making this assessment (Figure 1 and Figure 2). Segregation with disease status and familial data were also considered for one variation (p.Arg495Gln). All the novel variations and data evidencing disease association or absence of disease association are presented in Table 1 and Appendix 3. LTBP2 protein sequence alignments that allow assessment of evolutionary conservation are presented in Appendix 4. The position of candidate disease-influencing variations and previously reported mutations within the LTBP2 protein are presented in Figure 3. Some features of the variations observed in this study are described further.

**Figure 4:**
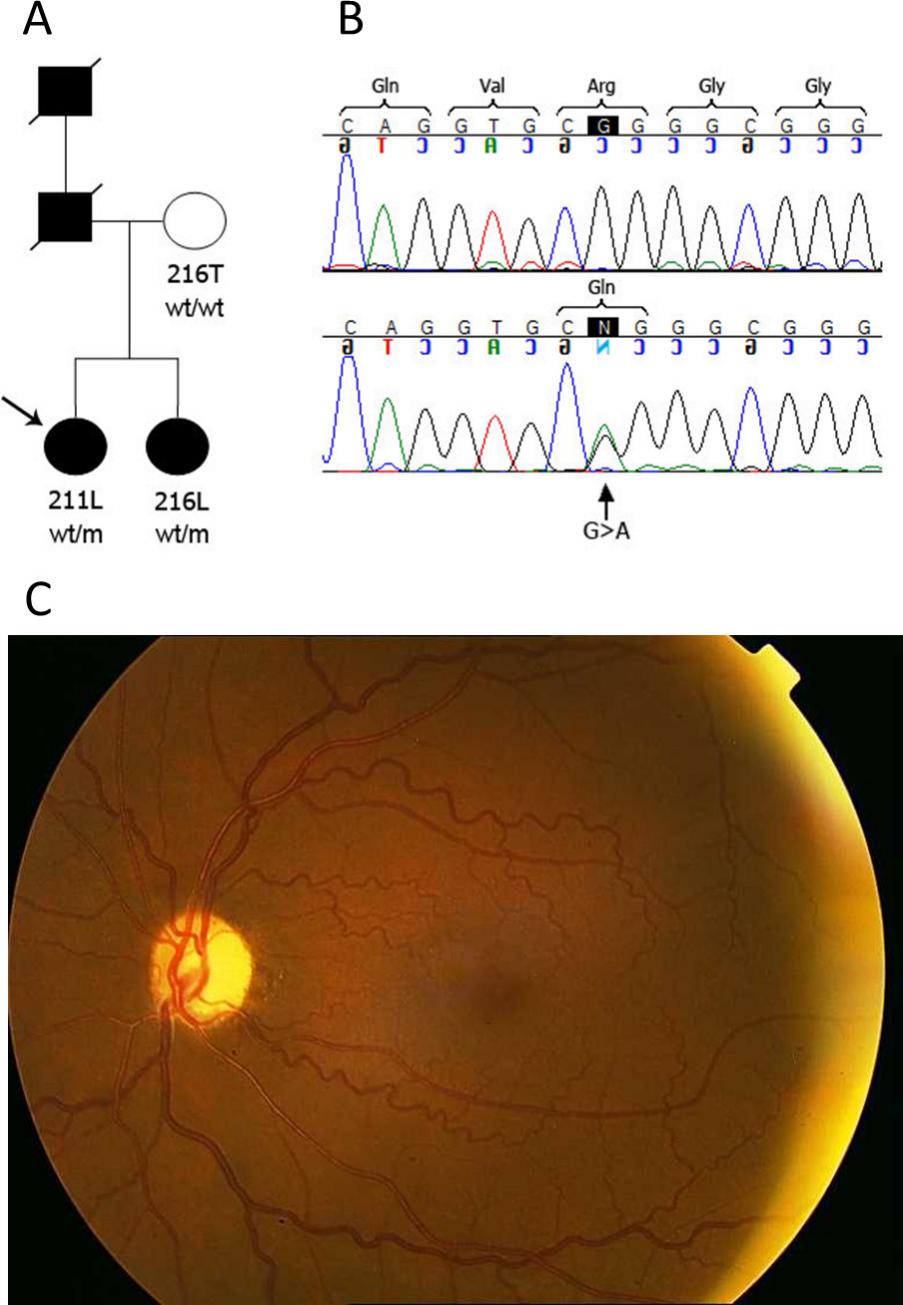
*LTBP2* mutation p.Arg495Gln in a family with primary open angle glaucoma. **A**: Pedigree of the family. 216T, 211L, and 216L are the ID numbers of living members of the family. Filled circles and squares: affected with glaucoma; open circles and squares: unaffected phenotype; wt=wild-type allele, m=mutated allele. **B**: Chromatograms showing homozygous wild-type *LTBP2* genotype c.1484G (top) and heterozygous mutated genotype c.1484G>A (bottom). **C**: Fundus image of the proband’s left eye. Diffuse optic disc atrophy, enlarged cup-to-disc ratio with notching of the neuroretinal rim in the inferior pole of the disc, and severe nerve fiber layer loss especially in inferior area are evident.

**Figure 5:**
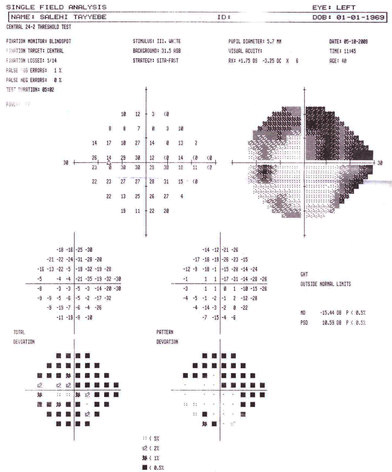
Visual field defects in the left eye of patient with POAG (211L). The single field analysis printout shows severe visual field loss with dense superior arcuate scotoma that threatens the fixation and inferior nasal field defect.

Five novel coding variations in *LTBP2* assessed as causing or as risk factors for disease were observed in five of the 42 patients with POAG screened (Table 1). Clinical features of the five patients are presented in Appendix 5. Age at onset in the patients harboring these putative POAG-contributing mutations ranged from 22 to 70 years. P.Pro432Leu among these was also observed in a patient with PEXG. The variation affecting p.Arg495Gln was observed in two siblings with POAG (Figure 4A,B). A fundus image of an eye of the proband showing features that confirm a diagnosis of glaucomatous optic neuropathy and results of perimetry showing visual field loss are presented in Figure 4C and Figure 5, respectively. Age at onset of the two sisters was 20 and 22 years. Furthermore, the deceased father and grandfather had had glaucoma and had become blind in one or both eyes in the third decade of their lives. Genetic analysis showed that the mother carried wild-type alleles, and clinical examination showed that she was free of ocular anomalies. The nucleotide sequence variation that caused p.Arg495Gln was not observed in 400 unaffected elderly ethnically matched control individuals. Interestingly, both siblings and also another POAG patient (213R) with an *LTBP2* mutation had the same cardiac anomalies that had previously been attributed to *LTBP2* mutations in WMS and MFS pedigrees [6]. The variation affecting p.Gln1417Arg was the only putative POAG-contributing variation found that occurred in the homozygous state (Table 1).The parents, who are deceased, were first cousins. Of the five patients who harbored the novel coding variations, all except the patient with the variation that caused p.Arg495Gln were apparently sporadic as they reported they were the only known individual with POAG in the respective families.

**Figure 6:**
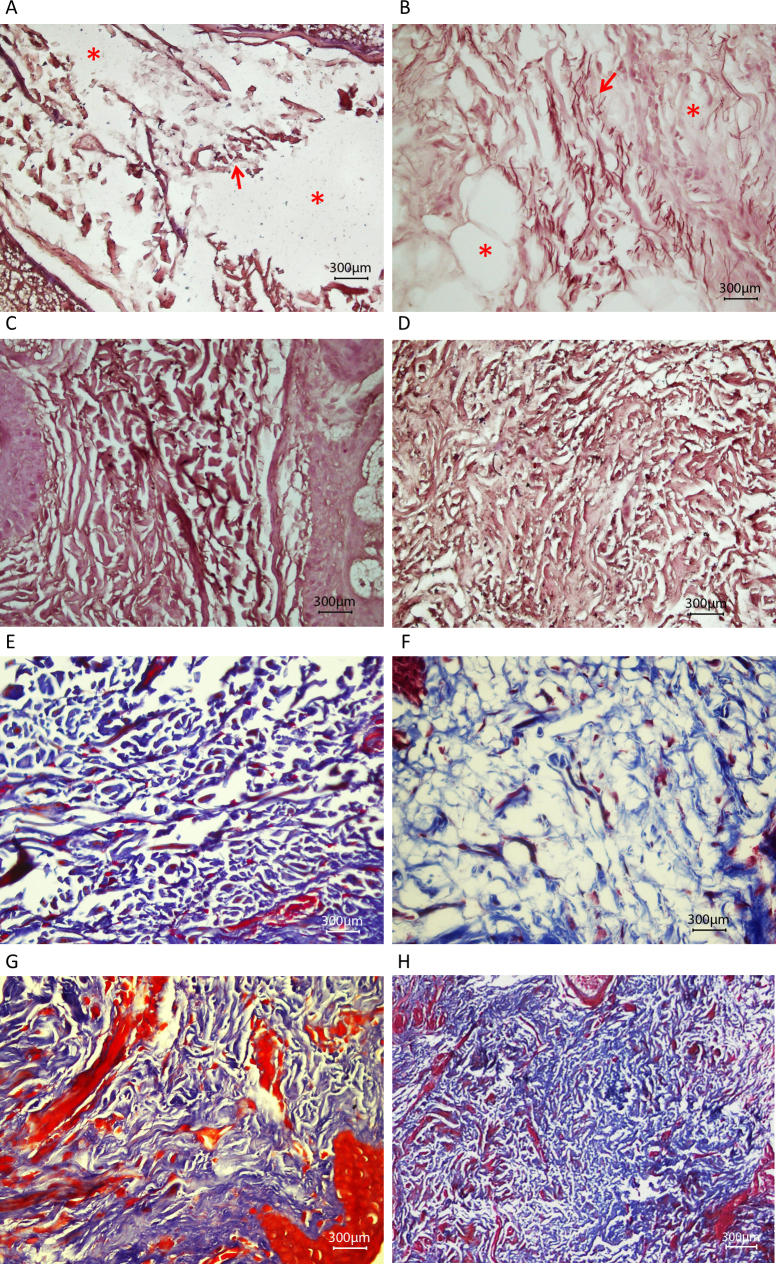
Light microscope images of skin tissue sections from patients with p.Tyr1792fsX55-causing mutations and age-matched control individuals. **A**–**D**: Orcein Geimsa–stained elastic fibers of a homozygous carrier with primary congenital glaucoma (PCG; **A**) and control individual (**C**), a heterozygous carrier with pseudoexfoliation (PEX) syndrome (**B**) and a control individual (**D**) visualized with a light microscope. Elastic fibers were sparser and fragmented in each patient compared to the respective control. Examples of fragmented fibers and sparse regions are shown, respectively, with arrows and * symbol. **E**–**H**: Trichrome stained collagen fibers of individual with PCG (**E**) and control individual (G), individual with PEX syndrome (F) and control individual (H) visualized with a light microscope. Collagen fibers, stained with blue, were sparser in each patient compared to the respective control. Arrector pili muscles appear red in G.

**Figure 7:**
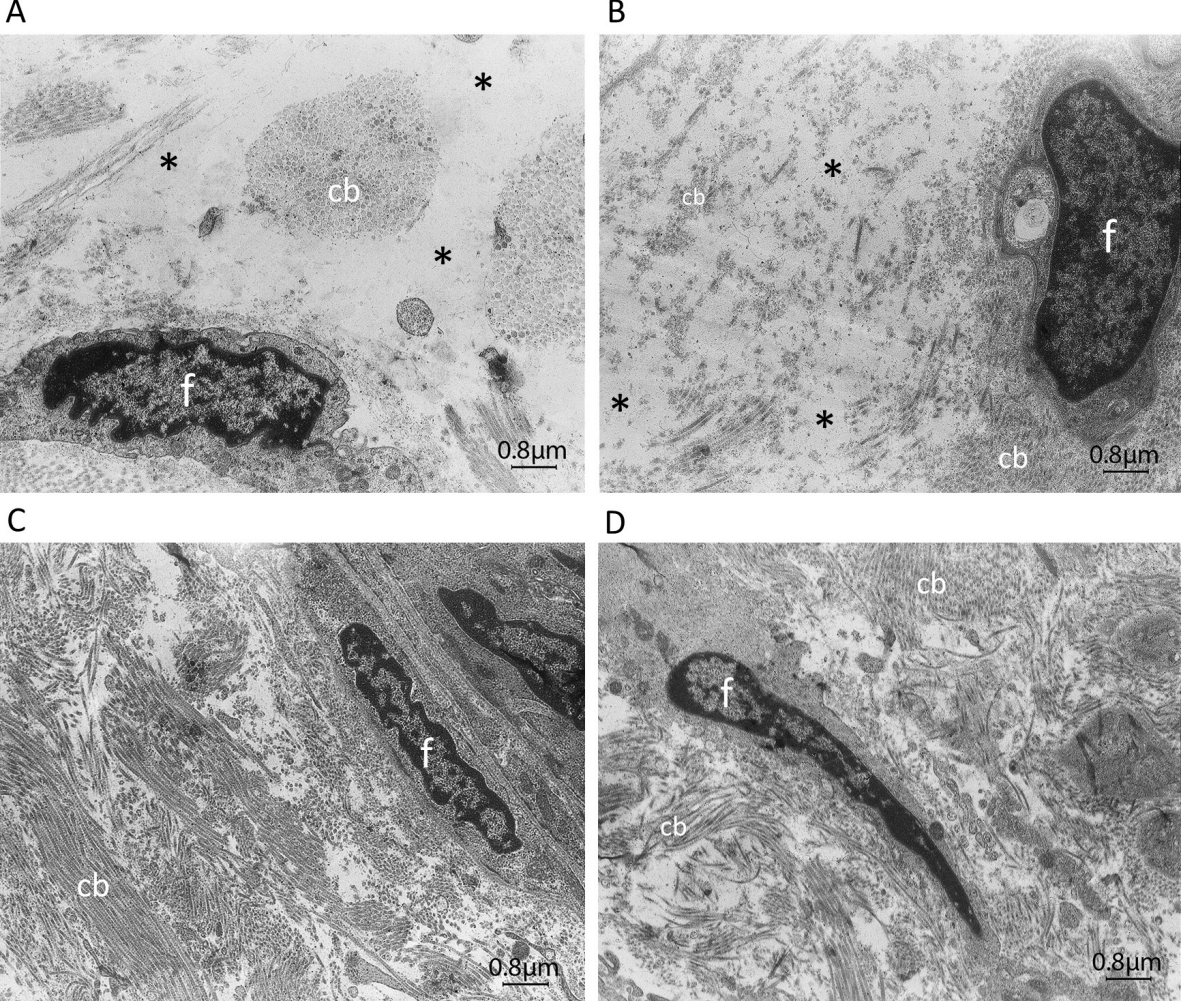
Transmission electron microscope images of skin tissue sections from patients with p.Tyr1792fsX55-causing mutations and age matched control individuals. Representative electron micrographs of a homozygous carrier with primary congenital glaucoma (PCG; **A**) and the control individual (**C**), and a heterozygous carrier with pseudoexfoliation (PEX) syndrome (**B**) and the control individual (**D**). More areas that appear devoid of extra cellular matrix (ECM) structures are evident in the image taken from the patients’ samples (**A**, **B**), and the ECM of the patient with PCG (A) is sparser than that of his older mother with PEX syndrome (**B**). Examples of sparse regions are shown with a star (∗). f, fibroblast; cb, collagen bundles.

**Figure 8:**
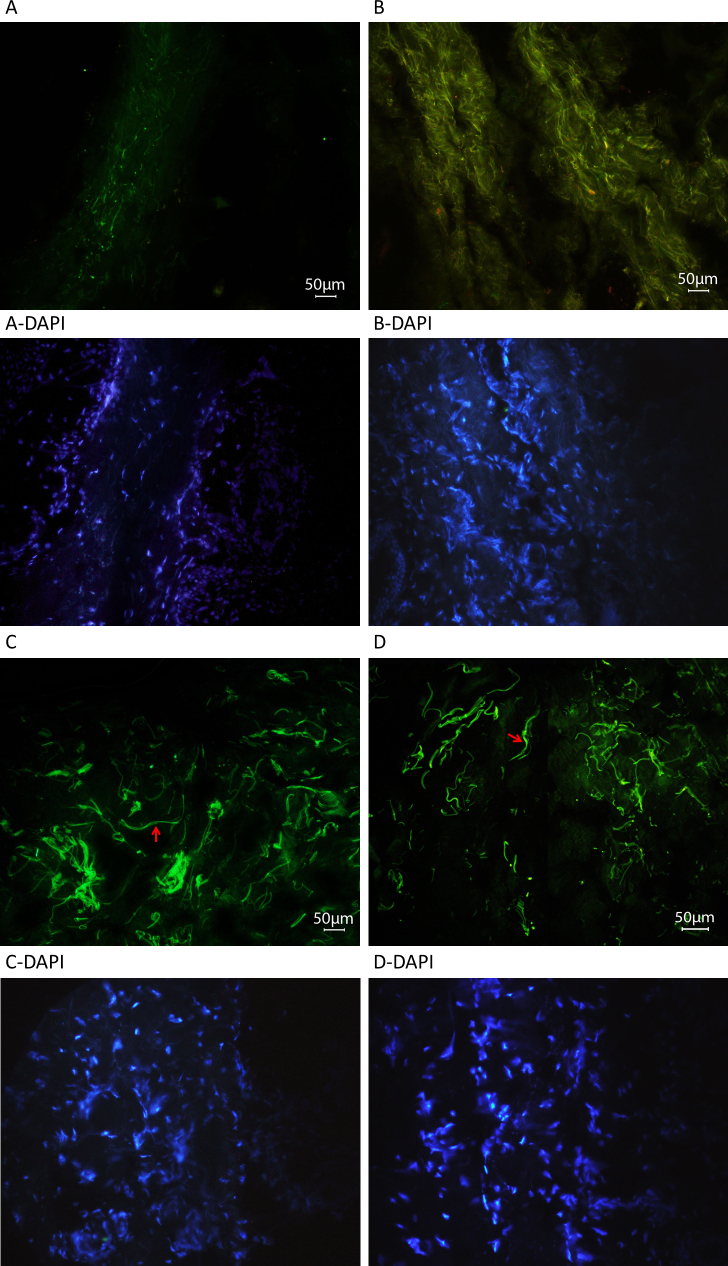
Fluorescent microscope images of skin tissue sections from patients with p.Tyr1792fsX55-causing mutations and age-matched control individuals stained for latent transforming growth factor-beta binding protein 2. Representative immunofluorescent cryosections from a homozygous carrier with primary congenital glaucoma (PCG; **A**) and the control individual (**C**), and from a heterozygous carrier with pseudoexfoliation (PEX) syndrome (**B**) and the control individual (**D**). Fibers stained for latent transforming growth factor-beta binding protein 2 (LTBP2) in the patient with PCG are thinner and noticeably fewer (**A**) compared with the control individual (**C**). Fibers in the patient with PEX syndrome are also thinner, but dense and convoluted (**B**) compared to the control individual (**D**). Examples of longer thicker fibers in the control sections are shown with arrows; these were not seen in the sections of patients’ tissues. Negative control is shown in Figure 9.

**Figure 9:**
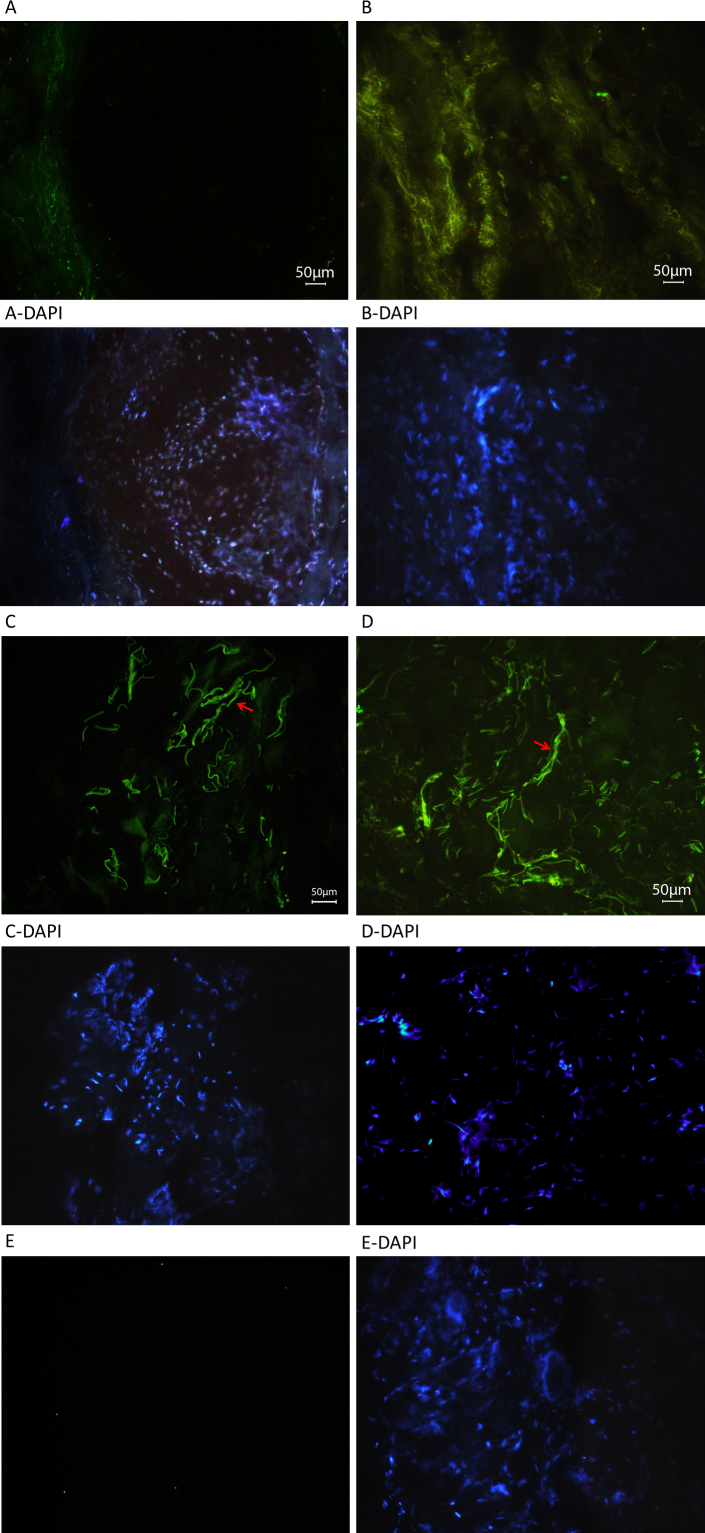
Fluorescent microscope images of skin tissue sections from patients with p.Tyr1792fsX55-causing mutations and age-matched control individuals stained for fibrillin-1. Representative immunofluorescent cryosections from a homozygous carrier with primary congenital glaucoma (PCG; **A**) and the control individual (**C**), and a heterozygous carrier with pseudoexfoliation (PEX) syndrome (**B**) and the control individual (**D**). The thick and long fibers that stained for fibrillin-1 in the control individuals (**C**, **D**; arrows) were not observed in multiple sections derived from the patient tissues (**A**, **B**). Fibers in the patient with PEX syndrome are dense and convoluted (**B**) compared to the control individuals (**D**). Negative control is shown in **E**.

**Figure 10:**
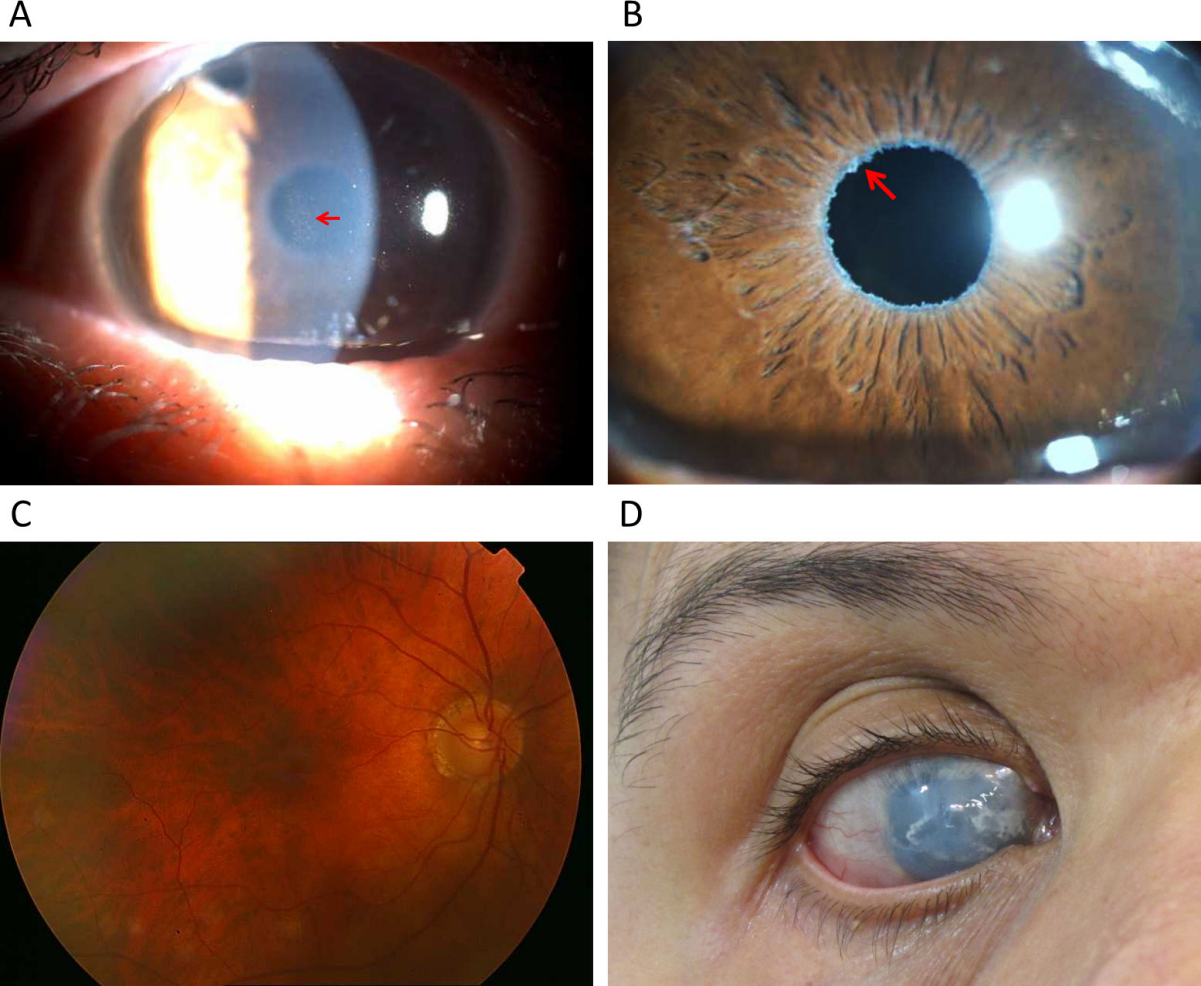
Images of eyes of a patient with pseudoexfoliation glaucoma syndrome and a patient with primary congenital glaucoma with heterozygous and homozygous p.Tyr1792fsX55-causing mutation in LTBP2, respectively. **A** and **B**: Images showing deposition of pseudoexfoliation material (arrows) on the endothelial surface of the cornea (arrow in **A**) and at the pupillary border of the iris (arrow in **B**) in the eye of an individual diagnosed with pseudoexfoliation glaucoma (PEXG) syndrome. **C**: Fundus photograph of the same PEXG individual exhibiting characteristic features of glaucomatous optic neuropathy. Diffuse neuroretinal rim thinning with more involvement of the inferior rim, notching and peripapillary atrophy all around the disc are evident. **D**: Image of an eye of an individual with PCG showing diffuse corneal opacity and calcific band keratopathy.

Among the 15 patients with PEX and 33 patients with PEXG, four novel coding variations were observed that were not found in controls (Table 1). The variations affecting p.Ile667Leu and p.Met1567Val, though novel and rare (not observed in 200 controls), are probably not associated with disease (Table 1). The variations that caused p.Pro432Leu and p.Tyr1792fsX55 likely contribute to the disease status of the patients with PEX syndrome. P.Pro432Leu is the variation also observed in a patient with POAG. The difference in the frequency of the causative variation in the patients (2/180 chromosomes) and the controls (0/800 chromosomes) is statistically highly significant (p=0.003). The patient with PEX syndrome who carried the p.Tyr1792fsX55-causing mutation in the heterozygous state was the mother of the proband (individual 21) of the PCG pedigree in which *LTBP2* was identified as a PCG causative gene [2]. Observation of PEX syndrome in a heterozygous carrier and PCG in a homozygous carrier of the same mutation was intriguing. Histology was performed to get some insight into the effects of the mutation and the differential effects in the two individuals. Skin tissue was used because access to ocular tissue was not feasible. Light, electron, and fluorescent microscopy shown abnormalities in the ECM of both patients (Figure 6, Figure 7, and Figure 8). In the light microscope images, the elastic fibers were sparser and fragmented in each patient compared to the respective control (Figure 6A–D). In general, disruption of the elastic fibers appeared more severe in the patient with PCG (Figure 6A) compared to his mother with PEX syndrome (Figure 6B), who was 55 years old and 25 years older than her son. Collagen fibers were also sparser in each patient compared to the respective control (Figure 6E–H). Finally, electron micrographs also clearly showed that the ECM of each patient was sparser than that of the controls (Figure 7A compared to Figure 7C, and Figure 7B compared to Figure 7D), and that the ECM of the patient with PCG was more severely disrupted than the ECM of the patient with PEX syndrome (Figure 7A compared to Figure 7B). Antibodies against LTBP2 and fibrillin-1-stained fibers that were longer and thicker in the ECM of the control individuals (Figure 8 and Figure 9). The fibers were sparse in the patient with PCG, but dense and convoluted in the ECM of the patient with PEX syndrome. This appearance is characteristic of microfibrils in patients with PEX syndrome [25]. The patterns described were consistently observed in multiple sections from the individuals studied. When the mother was examined in early 2011, she was diagnosed with PEX syndrome without glaucoma. Upon reexamination in 2012, her presentation had evolved into PEXG syndrome. Images of the eyes of the mother with PEXG syndrome and the son with PCG are shown in Figure 10. Depositions of PEX material and glaucomatous damage are evident in the mother’s eye.

## Discussion

Mutations in *LTBP2* have previously been shown to cause several disorders, all of which have ocular manifestations, and one of the ocular manifestations is often glaucoma [1-5]. Contrary to other known genes causing PCG (*CYP1B1*) or POAG (*MYOC*, *OPTN*, and *WDR36*), one can easily consider a plausible cellular and molecular basis for association between *LTBP2* and the glaucoma phenotype. LTBP2 is an extracellular matrix microfibril protein, and defects in the ECM of the trabecular meshwork may affect facility of aqueous fluid outflow resulting in increased IOP [26]. This notwithstanding, the consequences of *LTBP2* mutations for regulating TGF-β signaling may also be relevant to the etiology of glaucoma. Five putative disease-contributing or risk factor mutations in *LTBP2* were observed among 42 patients with POAG without *CYP1B1* and *MYOC* mutations. Although the size of the cohort screened was small, the frequency of the observed *LTBP2* mutations compares well with that of other known genes causing POAG [18,27]. Relevant to this finding, *ADAMTS10* was recently reported as a POAG causative gene in a canine model of the disease [28]. ADAMTS10 is a member of a disintegrin and metalloproteinase with thrombospondin motifs family of secreted proteases that similarly to LTBP2 is involved in the formation of the ECM [29]. Both proteins are expressed in the trabecular meshwork [2,28]. POAG rarely exhibits Mendelian inheritance, and many genetic and possibly non-genetic factors contribute to its pathogenesis. Taken together, the data presented here support the proposal that structural and/or functional defects of the ECM and/or microfibrils are among the factors that contribute to POAG and that mutations in genes coding for various components of the ECM and microfibrils including *LTBP2* are implicated in the disease’s etiology. ECM abnormalities have also previously been suggested to affect IOP elevation in glaucoma [30-33].

PEX syndrome is a fibrillinopathy that can present with numerous clinical complications [34]. Ocular tissues are most commonly affected. In addition to LTBP2 being a component of PEX material, there is increased TGF-β and LTBP2 in the aqueous fluid of affected individuals [25]. In addition, *LTBP2* and *FBN1* were identified as upregulated genes in anterior segment eye tissues of individuals with PEX [35]. The role of TGF-β1 in the pathogenesis of PEX has been emphasized, and it was suggested that LTBP2 via its effects on TGF-β activation may be involved in the disease process [25]. Variable incidence of PEX in different populations and increased risk in relatives of affected individuals indicate a genetic component in the disease’s etiopathogenesis, but knowledge of the genetics of PEX is rudimentary [22]. Association between some single nucleotide polymorphisms in *LOXL1* encoding lysyl oxidase-like 1 and PEX syndrome has been reported, although the PEX-associated variants in *LOXL1* exhibit low penetrance [36-38]. Lysyl oxidase-like 1 is a cross-linking enzyme with functions in elastic fiber formation and stabilization [39]. Here, we report the presence of two putative *LTBP2* mutations in patients with PEX. One mutation affected p.Pro432Leu and was observed in a patient with PEXG. The fact that it was also observed in a patient with glaucoma argues in favor of its pathogenicity. The second mutation caused p.Tyr1792fsX55 and was observed in a patient with PEXG; the same mutation in the homozygous state caused PCG in other family members. It was absent in 400 control individuals over the age of 60 without eye disorders. Penetrance of the p.Tyr1792fsX55-causing mutation regarding PEX is apparently incomplete because the father of the family who harbored the same mutation was diagnosed normal; he is now deceased. Histological analysis showed that the p.Tyr1792fsX55-causing mutation disrupted the ECM and microfibril structures, and that the disruptions were more severe in homozygous carriers of the mutation. Histological analysis in a patient with WMS who harbored a different mutation in *LTBP2* also showed disruption of the ECM and microfibril structures. These findings further support the contention that *LTBP2* mutations may contribute to various disorders with ocular manifestations by affecting the extracellular matrix.

There is variability in phenotypic consequences of mutations in *LTBP2*. A notable observation is that the mutation affecting p.Pro432Leu was observed in individuals with POAG and PEXG. The data presented here suggest that the penetrance of most observed *LTBP2* mutations is incomplete as disease in the patients usually appeared sporadically. This is consistent with the common classification of POAG and PEX syndrome as complex disorders. As both diseases are multifactorial, factors in addition to the *LTBP2* sequence variations affected disease status in the individuals harboring the mutation. Phenotypic presentations in individuals of the Roma/Gypsy population who harbored homozygous mutations causing p.Arg299X in *LTBP2* were reported to range from PCG with trabecular meshwork dysgenesis to Marfan syndrome-like zonular disease and late onset angle closure glaucoma [40]. In another family study, the same p.Arg299X mutation was reported to cause a recessive ocular syndrome exhibiting megalocornea, spherophakia, and secondary glaucoma [3]. Homozygous *LTBP2* mutations are likely to result in a more severe phenotype; homozygous mutations were reported to cause PCG and WMS, which are early onset diseases with more severe clinical manifestations than POAG and PEX syndrome, which are late onset diseases [1,2]. Notably, POAG and PEX are not considered recessively inherited diseases. Phenotypic variability associated with *FBN1* mutations is also evident. As stated, mutations in *FBN1* can cause simple ectopia lentis, MFS, WMS as well as other related disorders [41]. The mutation affecting p.Cys1223Tyr has been observed in patients with MFS [42] and Shprintzen-Goldberg syndrome [43]. The variations in phenotypic manifestations of mutations in the two related genes *LTBP2* and *FBN1* may be due to environmental, epigenetic, or stochastic events. More interesting, it is also likely that there are epistatic relationships between genes coding various components of the ECM. An intriguing possibility is that some ECM-related proteins may be able to compensate one another’s functions to different degrees.

In conclusion, we report that *LTBP2* mutations may cause or be risk factors for POAG and PEX. The findings emphasize the importance of the extracellular matrix to biologic functions affected in these disorders. POAG and PEX syndrome are common disorders important to public health issues of most populations. It is of paramount importance that larger patient cohorts with these diseases be screened for mutations in *LTBP2* and other related ECM protein coding genes to better quantify their contributions to the diseases.

## Appendix 1.

LTBP2 primers. To access the data, click or select the words “Appendix 1.”

## Appendix 2.

LTBP2 sequence variations observed among 90 POAG and PEX Syndrome patients. To access the data, click or select the words “Appendix 2.”

## Appendix 3.

Novel sequence variations in LTBP2. To access the data, click or select the words “Appendix 3.”

## Appendix 4.

Alignment of novel amino acid variations in LTBP2. To access the data, click or select the words “Appendix 4.”

## Appendix 5.

Clinical features of POAG patients with variations in LTBP2. To access the data, click or select the words “Appendix 5.”
